# Parent–child discrepancies in screening for Internet Gaming Disorder: Evidence from a clinical sample of Japanese adolescents

**DOI:** 10.1002/pcn5.70314

**Published:** 2026-03-08

**Authors:** Masaru Tateno, Takaki Shimode, Koki Ono, Ryotaro Shimomura, Eri Shiraishi, Kotaro Nanba, Yukie Tateno, Ayumi Takano

**Affiliations:** ^1^ Child and Adolescent Psychiatry, Tokiwa Child Development Center, Tokiwa Hospital Sapporo Japan; ^2^ Child Mental Health Clinic, Department of Neuropsychiatry Sapporo Medical University Hospital Sapporo Japan; ^3^ Child and Adolescent Psychiatry, Shimode Mental Clinic Hiragishi Branch Sapporo Japan; ^4^ Department of Preventive Medicine and Public Health Keio University School of Medicine Tokyo Japan; ^5^ Department of Drug Dependence Research National Institute of Mental Health, National Center of Neurology and Psychiatry Tokyo Japan

**Keywords:** behavioral addiction, Internet Gaming Disorder, parent–child discrepancy, screening

## Abstract

**Aim:**

Questionnaire‐based screening tools for Internet Gaming Disorder (IGD) are widely used in clinical and epidemiological research. However, discrepancies between child self‐reports and parent reports may complicate the interpretation of screening results, particularly when cutoff‐based classifications are applied.

**Methods:**

Participants were 58 adolescents (aged 10–18 years) attending child and adolescent psychiatry outpatient clinics and their parents. Gaming‐related problems were assessed using parallel screening instruments: the Internet Gaming Disorder Scale for Children (IGDS‐C) and the Parental version of the Internet Gaming Disorder Scale (PIGDS). Parent–child agreement was examined using dimensional analyses (Pearson's correlation), paired comparisons (paired *t*‐test with Wilcoxon signed‐rank test as a sensitivity analysis), and categorical agreement indices (concordance rate, Cohen's *κ*, and McNemar's test) based on the conventional cutoff score.

**Results:**

Parent‐ and child‐reported IGDS scores were moderately correlated (*r* = 0.61, *p* < 0.001), indicating substantial dimensional concordance. However, parents reported significantly higher IGDS scores than children (mean difference = −1.09, *p* < 0.001), a finding confirmed by the Wilcoxon signed‐rank test. Categorical agreement based on cutoff‐based screening classifications was low (*κ* = 0.16), with most discordant cases reflecting parent‐positive and child‐negative classifications. McNemar's test demonstrated a significant asymmetry in these discrepancies.

**Conclusion:**

Although parent and child IGDS scores demonstrate meaningful dimensional concordance, the application of fixed cutoff‐based screening classifications substantially reduces agreement, a pattern that may reflect differences in evaluative thresholds between informants. These findings highlight limitations of relying solely on self‐reported cutoff‐based measures and underscore the need for multi‐informant, dimensional approaches when interpreting IGD screening results in youth.

## INTRODUCTION

Digital games have become one of the most popular forms of recreation among children and adolescents worldwide.^1^, ^2^ With the widespread availability of the internet, online gaming has increasingly replaced offline, time‐limited forms of play. This transition has diversified game content and genres while simultaneously extending the amount of time young people spend engaged in gaming activities. Recent population‐based studies in Japan indicate that digital gaming has become highly prevalent among children and adolescents, with substantial proportions reporting prolonged gaming time in daily life. For example, a nationwide survey conducted in 2019 using a representative cohort of the Japanese population demonstrated widespread engagement in internet gaming among youth and young adults, reflecting contemporary gaming patterns in the post‐online gaming era.^3^ As a result, parental concerns about excessive gaming in children have become increasingly common.^4^

The evolution of gaming technology has further reinforced this shift.^5^ Early handheld gaming devices emphasized portability and individual ownership, marking a departure from shared family entertainment. Subsequent technological advances transformed these devices into platforms supporting multiple games and, eventually, online connectivity. With the widespread adoption of online connectivity, gaming has increasingly incorporated social interaction and structural features that may contribute to prolonged engagement and difficulty disengaging from play. With the emergence of networked gaming, play has increasingly become socially interactive and continuously updated, often lacking clear endpoints.^6^ Previous research has highlighted that features such as persistent social environments, in‐game social obligations, and continuous reward structures are associated with greater gaming involvement and increased risk of gaming‐related problems.^7^

Against this backdrop, Internet Gaming Disorder (IGD) was introduced in Section III of the DSM‐5 as a condition requiring further study, representing the first formal recognition of a behavioral addiction within the DSM framework.^8^ This proposal subsequently influenced the inclusion of Gaming Disorder as a mental disorder in the ICD‐11.^9^ The classification of excessive gaming behavior as a mental disorder has generated substantial international debate.^10^ While some scholars have emphasized the clinical relevance of gaming‐related problems, others have raised concerns that highly engaged but non‐problematic gaming behavior may be over‐pathologized. In particular, critics have argued that formal diagnostic recognition may obscure the distinction between pathological behavior and normative or adaptive high involvement in gaming.^11^, ^12^

While critics have warned against moral panic and diagnostic inflation, recent international validation studies have suggested that the ICD‐11 framework demonstrates improved diagnostic accuracy for newly defined addictive behaviors, including Gaming Disorder.^13^, ^14^

In Japan, the inclusion of Gaming Disorder in ICD‐11 received extensive media coverage and attracted considerable public attention.^15^ Consequently, many parents have come to interpret prolonged gaming as a potential mental health problem, often experiencing heightened anxiety when their children spend long hours playing games.^16^ In clinical settings, children and parents who present with gaming‐related concerns frequently display marked discrepancies in their perceptions of problem severity, with parents tending to view gaming behavior as more problematic than do the children themselves.^4^

Early identification of problematic gaming is widely considered important for prevention and timely intervention.^17^ Self‐report screening scales are commonly used for this purpose due to their practicality and efficiency.^3^, ^18^, ^19^ However, the assessment of gaming‐related problems in youth relies heavily on subjective reporting, raising the possibility that parent‐ and child‐reported evaluations may differ. Previous studies examining parent–child agreement in the assessment of IGD have reported moderate to high correlations between informants, suggesting substantial convergence at a dimensional level.^20^ Nevertheless, discrepancies may arise under certain conditions, such as differences in the observability of gaming behavior, concealed or private gaming activities, and divergent interpretations of functional impairment and problem severity. These factors may lead parents and children to apply different evaluative thresholds when judging whether gaming has become problematic.

The present study aimed to examine the degree of agreement between parent and child reports on screening questionnaires for gaming disorder. Gaming‐related problems were assessed using parallel screening instruments designed for children and parents, consisting of a child self‐report scale and a corresponding parent‐rated version.

## METHODS

### Participants

Participants were patients aged 10–18 years who attended the Tokiwa Child Development Center (Child and Adolescent Psychiatry clinic, Tokiwa Hospital) and the Shimode Mental Clinic Hiragishi Branch (Child and Adolescent Psychiatry clinic). Eligibility was determined based on a brief oral eligibility check conducted by the attending psychiatrist. Patients were asked whether they had played digital games for at least 1 h per week on average during the past week. This criterion was not intended as a screening threshold for problematic gaming, but rather as a pragmatic eligibility requirement to ensure that participants had at least minimal exposure to gaming behavior relevant to the study questionnaires. The question was asked directly to the child during the clinical interview, with caregivers present when appropriate. Among consecutively referred patients for whom both the child and a caregiver provided consent to participate were included in the study. Participant recruitment and data collection were conducted between April 2023 and June 2023.

Psychiatric diagnoses were made by board‐certified child psychiatrists according to DSM‐5 criteria, informed by clinical interviews, standardized psychological assessments, and evaluations by experienced clinical psychologists.

Patients were excluded if the attending psychiatrist judged that participation was not feasible due to the severity of psychiatric symptoms, acute psychiatric instability, or intellectual functioning that precluded reliable questionnaire completion.

### Study questionnaires

Participants and their parents were asked to complete a set of questionnaires independently. Both children and parents first provided background information, including age, sex, and average time spent gaming on weekdays and weekends. Subsequently, children completed self‐report questionnaires, while parents completed corresponding parent‐rated questionnaires assessing gaming‐related problems. Parents and children were instructed to complete the questionnaires separately to ensure independent reporting. Questionnaires were completed in the outpatient waiting area and typically required approximately 10 min to complete.

### Measures

The following questionnaires were used as study measures. Gaming‐related problems were assessed using parallel screening instruments designed for children and parents. Children were asked to complete the self‐report versions of the Internet Gaming Disorder Scale for Children (IGDS‐C). Parents or primary caregivers completed the corresponding parent‐rated versions, namely the Parental version of the Internet Gaming Disorder Scale (PIGDS). Japanese versions of both questionnaires were used, for which linguistic validity has been established by Takano et al.^21^, ^22^ These instruments are screening tools intended to assess the severity of gaming‐related problems in children and adolescents rather than to establish a clinical diagnosis.

### Internet Gaming Disorder Scale for Children

The IGDS‐C is a self‐report screening instrument based on the proposed diagnostic criteria for IGD in Section III of the DSM‐5.^22^, ^23^ The scale consists of nine items corresponding to the nine DSM‐5 IGD criteria, assessing core symptoms such as preoccupation, tolerance, withdrawal, loss of control, and functional impairment related to gaming. Each item is answered in a dichotomous format (yes/no), yielding a total score ranging from 0 to 9, with higher scores indicating greater IGD severity. Consistent with the DSM‐5–based IGD framework, a cutoff score of five or more endorsed criteria is commonly used to indicate a positive screening for IGD. In a comprehensive review comparing gaming disorder screening instruments, the IGDS (IGDS9‐SF: Internet Gaming Disorder Scale‐9 Short Form)^24^ was identified as one of the tools with the strongest overall coverage of DSM‐5 and ICD‐11 criteria.^18^ The reliability and validity of the Japanese version of the IGDS‐C have been established by Ono et al.^22^

### Parental version of the Internet Gaming Disorder Scale

The PIGDS^20^, ^25^ is a parent‐rated screening instrument designed to assess IGD symptoms in children and adolescents from the caregiver's perspective. Similar to the child self‐report version, the PIGDS is based on the nine proposed DSM‐5 criteria for IGD and consists of nine dichotomous items (yes/no), yielding a total score ranging from 0 to 9. Higher scores indicate greater severity of gaming‐related problems as perceived by parents. In accordance with the DSM‐5 IGD framework, a cutoff score of five or more endorsed criteria is commonly used to indicate a positive screening classification. The reliability and validity of the PIGDS have been demonstrated in previous studies, and the Japanese version has been linguistically validated.^21^

### Statistical analysis

The overall comparison of parent‐ and child‐reported IGDS scores was specified a priori as the primary analysis.

Descriptive statistics were calculated for parent‐ and child‐reported IGDS scores. Differences between male and female participants in continuous variables were examined using the Mann–Whitney *U* test, given the non‐normal distribution of the data. Differences between parent‐ and child‐reported gaming time were examined using the Wilcoxon signed‐rank test. Associations between parent‐ and child‐reported scores as continuous measures were examined using Pearson's correlation coefficient.

Differences in mean IGDS scores between parent and child ratings were examined using a paired *t*‐test, given the paired nature of the data. Effect size for the paired comparison was calculated using Cohen's *dz*. As a sensitivity analysis to assess robustness against distributional assumptions, a Wilcoxon signed‐rank test was also conducted, excluding pairs with zero differences. The effect size for the Wilcoxon test was calculated as *r* = *Z*/√*N*.

Agreement between parent and child classifications based on the conventional cutoff score (≥5 indicating a positive classification) was evaluated using the concordance rate and Cohen's kappa coefficient (*κ*) to quantify agreement beyond chance. To examine the directionality of disagreement between parent and child classifications, McNemar's test was performed based on the discordant pairs in the 2 × 2 contingency table.

Scatter plots were used to visually inspect the relationship between parent‐ and child‐reported IGDS scores, with child‐reported scores on the *x*‐axis and parent‐reported scores on the *y*‐axis, including the line of equality and cutoff reference lines.

All statistical tests were two‐tailed, and statistical significance was defined as *p* < 0.05. All statistical analyses were conducted using StatFlex version 7 (Artech Co., Ltd., Osaka, Japan).

### Ethics

This study was approved by the Ethics Committee of Tokiwa Hospital (TH‐230417). Written informed consent was obtained from parents or legal guardians, and assent was obtained from participating children and adolescents; participants aged 18 years or older provided their own written informed consent. The study procedures were carried out in accordance with the Declaration of Helsinki.

## RESULTS

### Participant characteristics

The sample consisted of 58 adolescents (mean age = 13.8 ± 2.6 years), the majority of whom were male. Demographic and clinical characteristics of the participants are shown in Table 1.

**Table 1:** Demographic and clinical characteristics of the participants (*n* = 58).

	*n*	Age	
Overall	58	13.8 ± 2.6	
Male	49	13.7 ± 2.7	*p* = 0.260
Female	9	14.6 ± 2.4	

*Note*: Values are presented as mean ± SD or *n* (%), as appropriate. Group differences among the three diagnostic categories were examined using one‐way analysis of variance (anova).

### Comparison of parent‐ and child‐reported IGDS scores

**Figure 1:**
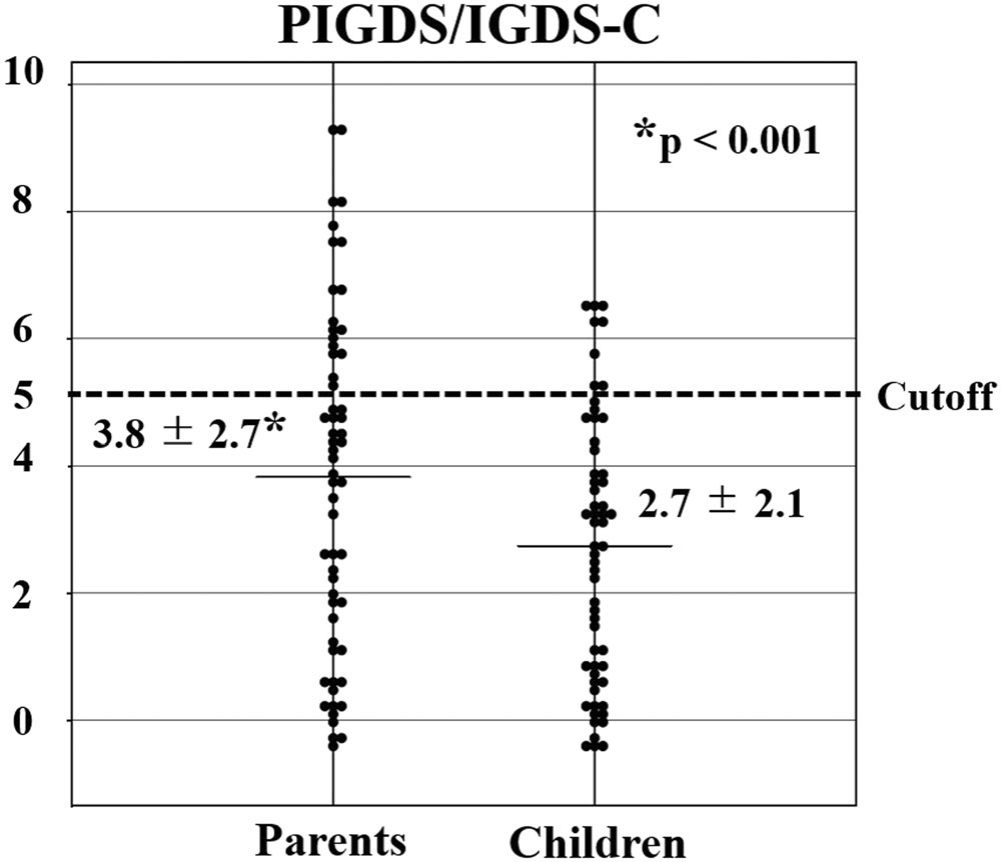
Comparison of parent‐ and child‐reported Internet Gaming Disorder Scale (IGDS) total scores. Paired comparison of parent‐reported (PIGDS) and child‐reported (IGDS‐C) total scores in the overall sample (*n* = 58). Each dot represents an individual participant. Horizontal bars indicate mean ± SD. The dashed line indicates the conventional cutoff score (≥5). Parent‐reported scores were significantly higher than child‐reported scores (paired *t*‐test, *p* ≤ 0.001).

Parent‐reported IGDS total scores were significantly higher than child‐reported IGDS‐C scores in the overall sample (parents: 3.8 ± 2.7; children: 2.7 ± 2.1; mean difference = −1.09, 95% CI: −1.65 to −0.52; paired *t*(57) = − 3.83, *p* < 0.001; Figure 1), corresponding to a medium effect size (Cohen's *dz* = 0.50). Parent‐ and child‐reported IGDS scores were moderately correlated (*r* = 0.61, *p* < 0.001), indicating that higher child‐reported scores were generally associated with higher parent‐reported scores.

A Wilcoxon signed‐rank test excluding pairs with zero differences (effective *N* = 49) yielded consistent results (*Z* = −3.43, *p* < 0.001), with a medium‐to‐large effect size (*r* = 0.52).

### Gaming time reported by parents and children

There were no significant differences between parent‐ and child‐reported gaming time on either weekdays or weekends (Table 2).

**Table 2:** Mean daily gaming time reported by parents and children.

	Gaming time (h/day)		
	Parents	Children	*p* value
Weekdays	3.8 ± 2.6	4.0 ± 3.2	*p* = 0.852
Weekends	5.6 ± 3.3	5.3 ± 3.4	*p* = 0.492

*Note*: Gaming time is presented as hours per day (mean ± SD). *p* values indicate paired comparisons between parent‐ and child‐reported gaming time (Wilcoxon signed‐rank test).

### Categorical agreement based on the conventional cutoff

When applying the conventional cutoff score (≥5), categorical agreement between parent‐ and child‐reported IGDS classifications was low (concordance rate = 63.8%; Cohen's *κ* = 0.16). Examination of the 2 × 2 contingency table revealed that most discordant classifications were parent‐positive and child‐negative (parent‐positive/child‐negative: *n* = 20; parent‐negative/child‐positive: *n* = 1), whereas concordant classifications included *n* = 4 parent–child positive and *n* = 33 parent–child‐negative cases. McNemar's test demonstrated a significant asymmetry in these discordant classifications (*χ* ^2^(1) = 15.43, *p* < 0.001), indicating that parents were significantly more likely than children to classify gaming‐related problems as positive. Consistent with these categorical findings, paired comparisons of continuous IGDS scores showed higher parent‐reported severity than child‐reported severity with a medium effect size (Cohen's *dz* = 0.50). Details of the cutoff‐based classification agreement are shown in Table S1.

## DISCUSSION

The present study provides a detailed examination of parent–child discrepancies in the assessment of IGD severity using a commonly applied questionnaire‐based screening approach. By integrating dimensional analyses, categorical agreement indices, and sensitivity analyses, the present findings offer important methodological insights into the interpretation of IGD screening results in youth.

At the dimensional level, parent‐ and child‐reported IGDS scores were moderately correlated, indicating that both informants generally recognized similar patterns of relative symptom severity. This suggests that parents and children are not describing fundamentally different behaviors, but rather share a common understanding of which individuals exhibit more severe gaming‐related problems. Such convergence at the continuous level is consistent with prior research on multi‐informant assessments of behavioral problems, where informants often agree on relative severity while differing in absolute judgments.^26^ Discrepancies between parent and child reports are well documented in child and adolescent psychiatry and are commonly observed in widely used multi‐informant instruments such as the Child Behavior Checklist, reflecting informant‐specific perspectives rather than measurement error.^27^

In contrast, categorical agreement based on the conventional cutoff score was low, highlighting a substantial divergence when cutoff‐based screening classifications were applied. Importantly, this disagreement was not random. The vast majority of discordant cases reflected parent‐positive and child‐negative classifications, a pattern that was supported by the results of McNemar's test. Furthermore, paired comparisons demonstrated that parents rated IGDS severity significantly higher than children, with a medium effect size. Importantly, the consistency between the paired *t*‐test and the Wilcoxon signed‐rank test indicates that the observed parent–child discrepancy in IGDS scores is robust to distributional assumptions and analytic approach. This discrepancy may reflect differences in evaluative thresholds or reporting tendencies when identifying gaming‐related problems, rather than being an artifact of statistical methodology.

This pattern of findings can be interpreted within theoretical frameworks emphasizing informant‐specific perspectives on behavioral problems. Children and adolescents tend to evaluate their behavior primarily through subjective experiences and perceived enjoyment, whereas parents are more likely to focus on observable functional consequences, such as academic difficulties, disrupted daily routines, or family conflict. As a result, parents may identify gaming‐related problems at an earlier stage, while children may normalize or minimize their behavior in the absence of perceived distress. When continuous symptom distributions are dichotomized using fixed cutoffs, these differences in evaluative thresholds become amplified, leading to low categorical agreement despite substantial dimensional concordance.

Consistent with prior work, gaming duration has frequently been examined as an indicator of risk for gaming‐related problems and has been incorporated into several screening and assessment frameworks. For example, ICD‐11–based approaches such as the GAMES Test include weekday gaming time as a component of risk classification, reflecting evidence that longer gaming time is associated with a higher likelihood of meeting criteria for Gaming Disorder.^3^ Population‐based and clinical studies have likewise reported associations between extended gaming time and increased risk of problematic gaming or related psychosocial difficulties.^17^, ^18^ However, prior literature has also emphasized that gaming duration alone does not necessarily capture the functional impairment or subjective distress that characterizes disordered gaming.

In the present study, parent‐ and child‐reported gaming time did not differ significantly, despite clear discrepancies in perceived gaming‐related problems. This pattern suggests that parent–child disagreement in screening outcomes may not be explained solely by differences in estimated gaming duration, but may instead relate to divergent interpretations of the functional impact and problematic nature of gaming behavior. In other words, the observed discrepancies in IGDS scores may reflect divergent evaluative thresholds regarding when gaming becomes problematic, rather than disagreements about gaming duration itself. This distinction underscores the limitation of relying solely on gaming time as an indicator of problem severity and highlights the importance of assessing functional impairment and subjective concerns when interpreting screening results.

Taken together, these findings suggest that while parent and child reports of gaming‐related problems show meaningful convergence at a dimensional level, consistent discrepancies emerge when fixed cutoff‐based classifications are applied. This highlights the importance of integrating dimensional severity, functional impairment, and multiple informant perspectives when interpreting questionnaire‐based screening results in clinical and research settings.

From a methodological perspective, dichotomizing continuous symptom measures using fixed cutoffs may obscure meaningful variation and inflate apparent disagreement between informants.^28^ Our findings therefore support calls for assessment frameworks that integrate dimensional severity, functional impairment, and multiple informants, particularly when questionnaire data are used to estimate prevalence or to identify individuals for further clinical evaluation.

Several limitations should be noted. The use of a single questionnaire limits conclusions about clinical diagnosis, and the cross‐sectional design precludes inferences about developmental changes in informant discrepancies over time. In addition, because participation required consent from both children and parents, the sample may not be fully representative of all consecutively referred patients. Future studies incorporating clinical interviews, standardized measures of functional impairment, and longitudinal designs will be essential for clarifying how parent–child discrepancies relate to clinical outcomes and help‐seeking behavior.

These findings suggest that, while parent and child IGDS scores covary meaningfully at a dimensional level, parents consistently rate gaming‐related problems as more severe than children, resulting in low cutoff‐based agreement. These discrepancies may reflect differences in evaluative thresholds rather than a lack of shared understanding, highlighting the need for cautious interpretation of screening results and for multi‐informant, dimensional approaches in the assessment of IGD in youth.

## CONCLUSION

In summary, parent and child reports of IGDS show meaningful concordance at a dimensional level, while agreement based on cutoff‐based screening classifications remains low in a clinical adolescent sample. Parents consistently rated gaming‐related problems as more severe than children, which may reflect differences in evaluative thresholds rather than random disagreement. These findings highlight an important limitation of relying solely on self‐reported, cutoff‐based screening results when assessing gaming‐related problems in youth. Questionnaire‐based screening tools are valuable for identifying individuals who may require further evaluation; however, their interpretation should incorporate multiple informants and consider dimensional severity and functional impairment. Integrating parent and child perspectives may improve the clinical utility of IGD screening and help avoid both underestimation and over‐pathologization of gaming behavior in adolescents.

## AUTHOR CONTRIBUTIONS

Masaru Tateno and Ayumi Takano developed the conception and design of the study. Masaru Tateno, Koki Ono, and Ayumi Takano created the study questionnaire. Masaru Tateno and Ayumi Takano obtained funding. Masaru Tateno, Takaki Shimode, Ryotaro Shimomura, Eri Shiraishi, Kotaro Nanba, and Yukie Tateno collected the data. Masaru Tateno contributed to the analysis and interpretation of data. Masaru Tateno and Takaki Shimode drafted the article. All authors contributed to the revision and approved the final version of the article. All authors had full access to the data in this study and take responsibility for the integrity of the data and the accuracy of the data analysis.

## CONFLICT OF INTEREST STATEMENT

The authors declare no conflicts of interest.

## ETHICS APPROVAL STATEMENT

The Ethics Committee of Tokiwa Hospital approved this study (TH‐230417). Written informed consent was obtained from parents or legal guardians, and assent was obtained from participating children and adolescents; participants aged 18 years or older provided their own written informed consent. This study was conducted in accordance with the principles of the Declaration of Helsinki.

## PATIENT CONSENT STATEMENT

N/A.

## CLINICAL TRIAL REGISTRATION

N/A.

## Supporting information

Supporting Information.

## Data Availability

The data that support the findings of this study are available from the corresponding author, Masaru Tateno, upon reasonable request. Requests will be considered on an individual basis based on ethical considerations and study requirements.
