# Supplementary material for: Parent–child discrepancies in screening for Internet Gaming Disorder: Evidence from a clinical sample of Japanese adolescents
Source: PCN Rep. 2026 Mar 8;5(1):e70314. doi: 10.1002/pcn5.70314 (PMC12967513; doi:10.1002/pcn5.70314)
Supplement: Supplementary file 1 — Supporting Information. [file PCN5-5-e70314-s001.docx]

|  | Parent positive | Parent negative | Total |
| --- | --- | --- | --- |
| Child positive | 4 | 1 | 5 |
| Child negative | 20 | 33 | 53 |
| Total | 24 | 34 | 58 |

Supplementary Table 1.

**Agreement between parent- and child-reported IGD classifications based on the conventional cutoff (≥ 5)**

This table shows the agreement between parent- and child-reported classifications of Internet Gaming Disorder (IGD). Frequencies are presented for concordant and discordant classifications between parents and children.
